# Characteristics of Extended-Spectrum β-Lactamase–Producing *Escherichia coli* From Dogs and Cats Admitted to a Veterinary Teaching Hospital in Taipei, Taiwan From 2014 to 2017

**DOI:** 10.3389/fvets.2020.00395

**Published:** 2020-07-16

**Authors:** Yi-Hsuan Huang, Nan-Ling Kuan, Kuang-Sheng Yeh

**Affiliations:** ^1^Department of Veterinary Medicine, School of Veterinary Medicine, College of Bioresources and Agriculture, National Taiwan University, Taipei, Taiwan; ^2^Biology Division, Animal Health Research Institute, New Taipei City, Taiwan; ^3^National Taiwan University Veterinary Hospital, Taipei, Taiwan

**Keywords:** extended-spectrum-β-lactamases, *Escherichia coli*, CTX-M, multilocus sequence typing, multidrug resistance

## Abstract

Extended-spectrum β-lactamases (ESBLs) are enzymes that mediate resistance to newer β-lactam antibiotics, including extended-spectrum cephalosporins and monobactams. The production of ESBL is primarily plasmid mediated, and such plasmids often comprise the genes that encode resistance to other classes of antimicrobials, such as aminoglycosides and fluoroquinolones. Therefore, ESBL-producing microorganisms leave clinicians with limited therapeutic options in both human and veterinary medicine. Compared with human medicine, information regarding ESBL-producing microorganisms is limited in veterinary medicine. We screened for ESBL-producing *Escherichia coli* in dogs and cats admitted to National Taiwan University Veterinary Hospital, Taipei, from 2014 to 2017 and further analyzed the genotypes and phylogenetic traits of these ESBL producers. Double disk tests specified by the Clinical and Laboratory Standards Institute were performed on 283 *E. coli* isolates and revealed a total of 65 *E. coli* (54 from dogs and 11 from cats) with the ESBL phenotype (22.8%). *bla*_CTX−M−1_ _group_ and *bla*_CTX−M−2group_ were the most commonly identified ESBL gene groups. *bla*_CTX−M−55_ was the main ESBL gene within the *bla*_CTX−M−1group_, whereas the *bla*_CTX−M−2group_ contained only *bla*_CTX−M−124_. The ESBL-producing *E. coli* were all resistant to ampicillin. The resistance rate to ceftiofur, doxycycline, enrofloxacin, and ciprofloxacin was 93.8, 73.8, 80, and 78.5%, respectively. Of the antibiotics tested, greater sensitivity to imipenem and gentamicin was noted. Multilocus sequence typing indicated that ST457, ST131, and ST648 were the most common sequence types. Our study identified eight ST131/O25b isolates, which is a global zoonotic clone of public health concern. The major ESBL genes of these clones were *bla*_CTX−M−174_ and *bla*_CTX−M−194_. Because companion animals such as dogs and cats are in close contact with humans, the characterization of ESBL producers originating from them is crucial from the perspective of both public health and veterinary medicine.

## Introduction

*Escherichia coli*, a type of Gram-negative bacteria is a ubiquitous inhabitant of the gastrointestinal tract of both humans and animals. This microorganism frequently causes urinary tract, skin, or soft tissue infections in cats and dogs (1). Commonly prescribed medications to treat *E. coli* infection in companion animals include ampicillin, amoxicillin-clavulanic acid, fluoroquinolones, or cephalosporins. However, the emergence of drug-resistant bacteria encountered in clinical practice decreases the therapeutic efficacy of these antimicrobial agents. One major mechanism of this drug resistance is the production of enzymes by microbes to inactivate antimicrobial agents. For example, β-lactam agents are widely used to treat bacterial infections in veterinary medicine, whereas extended-spectrum β-lactamases (ESBLs) are a group of enzymes that mediate resistance to most β-lactam antibiotics, including extended-spectrum cephalosporins and monobactams but excluding carbapenems and cephamycins (2). ESBLs are inhibited by clavulanic acid, sulbactam, and tazobactam; this fact is used as a criterion to classify β-lactamases and for ESBL diagnosis purposes (3). TEM, SHV, and CTX-M-group enzymes are examples of commonly encountered ESBLs (2). ESBL producers usually exhibit a multi-drug-resistant phenotype. In addition, the ESBL genes are mainly plasmid mediated, thus facilitating the transmission of drug-resistant genes to other bacteria. Such a situation poses a challenge for infection management in clinical practice. ESBLs have been previously documented primarily in human clinical cases (4). Because companion animals such as dogs and cats are in close contact with humans, they could contract ESBL-producing microorganisms from humans and then possibly transmit them back to humans, which represents a public health concern (5).

Information regarding the prevalence of ESBL producers or the genotypes of these clinical isolates from cats and dogs is limited in Taiwan. It is imperative to investigate related matters from both a veterinary medicine and public health perspective (6). The present study analyzed a collection of *E. coli* isolates obtained from National Taiwan University Veterinary Hospital (NTUVH), a university-based veterinary teaching hospital in Taipei, from 2014 to 2017 to determine the prevalence of ESBL-producing *E. coli*, assess their antimicrobial profile, and characterize the strains phylogenetically through multilocus sequence typing (MLST). The results obtained should provide insights into the role of ESBL-producing *E. coli* in companion animals. Some of the data herein have previously been reported at a conference (7).

## Materials and Methods

### Sample Collection

NTUVH is a teaching hospital affiliated with the College of Bioresources and Agriculture at National Taiwan University located in Taipei, Taiwan. Between 2014 and 2017, 283 *E. coli* isolates obtained from dogs (*n* = 224) and cats (*n* = 59) that were admitted to NTUVH were screened for ESBL producers. These *E. coli* isolates were cultured from different sources of the animals and identified using a Vitek 2 Compact (Biomérieux, Marcy-I'Etoile, France) to the species level and stored at −80°C. Urine and pus samples from the uterus or wounds comprised almost 70% (47 and 22%, respectively) of the *E. coli* sources. These samples were collected from the animals to facilitate diagnosis and treatment. An ethical review was not required for this study.

### ESBL Phenotype Testing

The ESBL producers of *E. coli* were tested using combination disk tests with cefotaxime and ceftazidime (30 μg), with and without clavulanic acid (10 μg), as specified by the Clinical and Laboratory Standards Institute (8). Briefly, the tested *E. coli* were plated on Muller–Hinton agar at a concentration of 0.5 McFarland standards and incubated at 35°C for 16–18 h. A difference of 5 mm or more in the inhibition zones for either cefotaxime or the ceftazidime–clavulanic acid combination vs. the corresponding cefotaxime or ceftazidime alone was defined as an ESBL-producing *E*. *coli*. *Klebsiella pneumoniae* ATCC 700603 and *E. coli* ATCC 25922 were used as the positive and negative controls, respectively.

### Detection of *bla* Genes

The *E. coli* isolates that were phenotypically ESBL producers were analyzed using polymerase chain reaction (PCR) to detect their *bla* genes. Bacterial DNA was extracted using the boiling method (9). Briefly, bacterial strains were cultured overnight at 37°C on tryptic soy agar plates (Difco/Becton Dickinson, Franklin Lakes, NJ), and a loopful of cells was boiled in 200 μL of ddH_2_O for 10 min. The supernatant was saved after centrifugation at 12,000 × g for 10 min and used as the source of template DNA for PCR. The primers used to amplify *bla*_CTX−M−1−group_, *bla*_CTX−M−2−group_, *bla*_CTX−M−8−group_, *bla*_CTX−M−9−group_, *bla*_CTX−M−25−group_, *bla*_SHV_, *bla*_TEM_, and the expected PCR product sizes are listed in Table 1. The PCR cycling conditions were as follows: initial denaturation at 95°C for 5 min, followed by 35 cycles at 95°C for 30 s, annealing at 52–55°C (as specified in Table 1) for 30 s, and a 72°C extension for 1 min. Ten microliters of each PCR sample were loaded onto a 1.5% agarose gel and electrophoresed at 100 V for 30 min. The gels were then stained with a fluorescent nucleic acid dye (Biotium, Hayward, CA) and examined under ultraviolet illumination. The PCR products were then purified using a GeneJet PCR purification kit (Thermo Fisher Scientific, Waltham, MA) according to the protocol provided by the manufacturer and subjected to sequencing (Mission Biotech, Taipei, Taiwan). The DNA sequences were examined using the Beta-Lactamase DataBase (www.bldb.eu) (17).

**Table 1:** Sequences of primers used in this study.

**PCR target**	**Primer**	**Sequences (5′-3′)**	**Annealing Tm (°C)**	**Predicted PCR size (bp)**	**References**
*bla*_TEM_	TEM-F	TCGGGGAAATGTGCGCG	55	972	(10)
	TEM-R	TGCTTAATCAGTGAGGCACC			
*bla*_SHV_	SHV-F	GCCTTTATCGGCCCTCACTCAA	54	819	(11)
	SHV-R	TCCCGCAGATAAATCACCACAATG			
*bla*_CTX−M−1−group_	CTX-M-1-F	CCCATGGTTAAAAAATCACTGC	54	942	(12)
	CTX-M-1-R	CAGCGCTTTTGCCGTCTAAG			
*bla*_CTX−M−2−group_	CTX-M-2-F	CGACGCTACCCCTGCTATT	52	552	(13)
	CTX-M-2-R	CCAGCGTCAGATTTTTCAGG			
*bla*_CTX−M−8−group_	CTX-M-8-F	TCGCGTTAAGCGGATGATGC	52	666	(13)
	CTX-M-8-R	AACCCACGATGTGGGTAGC			
*bla*_CTX−M−9−group_	CTX-M-9-F	ATGGTGACAAAGAGAGTGCAAC	55	876	(14)
	CTX-M-9-R	TTACAGCCCTTCGGCGATGATT			
*bla*_CTX−M−25−group_	CTX-M-25-F	GCACGATGACATTCGGG	52	327	(13)
	CTX-M-25-R	AACCCACGATGTGGGTAGC			
*adk*	adk-F	ATTCTGCTTGGCGCTCCGGG	54	583	(15)
	adk-R	CCGTCAACTTTCGCGTATTT			
*fumC*	fumC-F	TCACAGGTCGCCAGCGCTTC	54	806	(15)
	fumC-R	GTACGCAGCGAAAAAGATTC			
*gyrB*	gyrB-F	TCGGCGACACGGATGACGGC	60	911	(15)
	gyrB-R	ATCAGGCCTTCACGCGCATC			
*icd*	icd-F	ATGGAAAGTAAAGTAGTTGTTCCGGCACA	54	878	(15)
	icd-R	GGACGCAGCAGGATCTGTT			
*mdh*	mdh-F	AGCGCGTTCTGTTCAAATGC	60	932	(15)
	mdh-R	CAGGTTCAGAACTCTCTCTGT			
*purA*	purA-F	CGCGCTGATGAAAGAGATGA	54	816	(15)
	purA-R	CATACGGTAAGCCACGCAGA			
*recA*	recA-F	CGCATTCGCTTTACCCTGACC	58	780	(15)
	recA-R	TCGTCGAAATCTACGGACCGGA			
*pabB*	O25pabBspe.F	TCCAGCAGGTGCTGGATCGT	65	347	(16)
	O25pabBspe.R	GCGAAATTTTTCGCCGTACTGT			
*trpA*	trpA.F	GCTACGAATCTCTGTTTGCC	65	427	(16)
	trpA2.R	GCAACGCGGCCTGGCGGAAG			

### Antibiotic Susceptibility Test

The ESBL-producing *E. coli* isolates were tested for susceptibility to antimicrobial agents used in clinical settings using the standard Kirby–Bauer disk diffusion method (8). The antimicrobial agents tested included β-lactams (amoxycillin/clavulanic acid, ampicillin, imipenem, and ceftiofur), tetracyclines (doxycycline), quinolones (enrofloxacin and ciprofloxacin), aminoglycosides (gentamicin), and sulfonamides (sulfamethoxazole/trimethoprim). The isolates were classified as susceptible, intermediate resistant, or resistant to the antimicrobial agents.

### Genotyping and Phylogenetic Analysis

The ESBL-producing *E. coli* strains were genotyped using MLST (15). Internal fragments of *adk, fumC, gyrB, icd, mdh, purA*, and *recA* were amplified through a PCR by using the primers listed in Table 1 and sequenced. They were then uploaded to the EnteroBase MLST website (http://enterobase.warwick.ac.uk/) for comparison. Phylogenetic analysis of the strains was performed using BioNumerics version 7.0 (Applied Maths, Sint-Martens-Latem, Belgium).

### *E. coli* ST131 O25b Detection

The PCR-based detection of *E. coli* ST131/O25b was based on the method described by Clermont et al. (16). The *trpA* and *pabB* primers and annealing temperature used are listed in Table 1. The PCR cycling conditions were as follows: initial denaturation at 94°C for 4 min, followed by 30 cycles at 94°C for 5 s, annealing at 65°C for 10 s, and 72°C extension for 5 min. Ten microliters of each PCR sample was loaded onto 2.0% agarose gel and electrophoresed at 100 V for 30 min. The gels were then stained with a fluorescent nucleic acid dye (Biotium) and examined under ultraviolet illumination.

## Results

A total of 283 *E. coli* isolates (59 from cats and 224 from dogs) were obtained during our study period (2014–2017). Table 2 lists the prevalence of ESBL-producing *E. coli* from dogs and cats. In total, 65 ESBL-producing *E. coli* isolates, 54 from dogs and 11 from cats, were acquired from our assay. The prevalence of ESBL-producing *E. coli* isolates was 24.1% (54/224) in dogs and 18.6% (11/59) in cats, and the total prevalence for both animals was 23.0% (65/283).

**Table 2:** Prevalence of ESBL-producing *E. coli* in dogs and cats.

	**2014**	**2015**	**2016**	**2017**	**Total**
Number of ESBL^+^ cat	4	1	3	3	11
Number of ESBL^+^ dog	18	14	15	7	54
Number of ESBL^−^ dog/cat	99	56	34	29	218
Total number assayed	121	71	52	39	283
ESBL prevalence	18.2%	21.1%	34.6%	25.6%	23.0%

Table 3 lists the distribution of *bla* genes from the 65 ESBL-producing *E. coli* isolates. *bla*_CTX−M−55_ of the *bla*_CTX−M−1group_ was the most prevalent *bla* gene encountered. The *bla*_CTX−M−2group_ contained only *bla*_CTX−M−124_. The *bla*_CTX−M−9group_ contained eight *bla* gene types, and *bla*_CTX−M−214_ was the most frequently observed. *bla*_TEM−215_ was the most common type encountered in the *bla*_TEMgroup_. We only detected *bla*_SHV−199_ in the *bla*_SHV_ group. We did not detect *bla*_CTX−M−8group_ or *bla*_CTX−M−25group_.

**Table 3:** Distribution of *bla* genes in the 65 ESBL-producing *E. coli* isolates.

***bla*_**CTX-M-1 GROUP**_**	***bla*_**CTX-M-2 GROUP**_**	***bla*_**CTX-M-9 GROUP**_**	***bla*_**TEM GROUP**_**	***bla*_**SHV GROUP**_**	***bla*_**CTX-M-8 AND CTX-M-25 GROUP**_**
*bla*_CTX−M−55_ (*n* = 24)	*bla*_CTX−M−124_ (*n* = 12)	*bla*_CTX−M−24_ (*n* = 1)	*bla*_TEM−81_ (*n* = 1)	*bla*_SHV−199_ (*n* = 4)	None
*bla*_CTX−M−69_ (*n* = 3)		*bla*_CTX−M−67_ (*n* = 1)	*bla*_TEM−215_ (*n* = 16)		
*bla*_CTX−M−194_ (*n* = 7)		*bla*_CTX−M−148_ (*n* = 1)	*bla*_TEM−219_ (*n* = 2)		
*bla*_CTX−M−199_ (*n* = 1)		*bla*_CTX−M−174_ (*n* = 4)	*bla*_TEM−226_ (*n* = 1)		
*bla*_CTX−M−211_ (*n* = 3)		*bla*_CTX−M−196_ (*n* = 1)	*bla*_TEM−230_ (*n* = 5)		
		*bla*_CTX−M−198_ (*n* = 1)			
		*bla*_CTX−M−214_ (*n* = 11)			
		*bla*_CTX−M−223_ (*n* = 1)			

**Figure 1:**
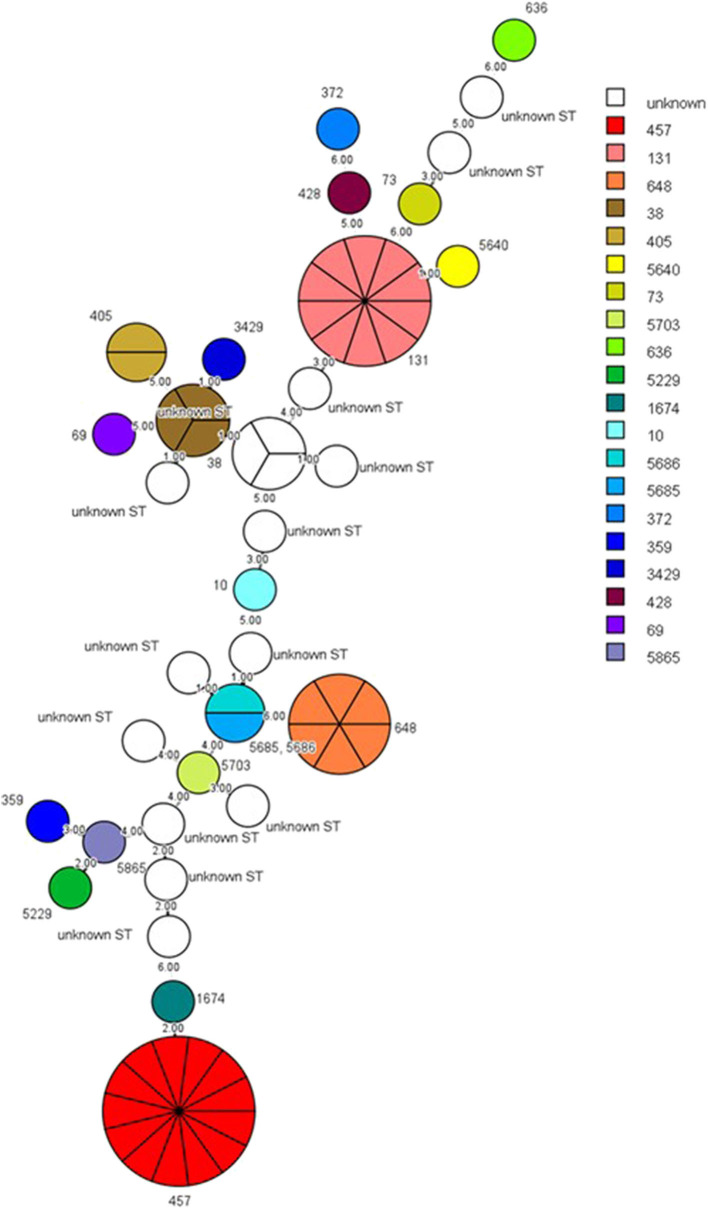
Minimal spanning tree of ESBL-producing *E. coli*. Each circle indicates one ST, subdivided into one sector for each isolate, and bordered by the ST number. White circles or sectors without an ST number denote a lack of comparison standard in the current databank. The numbers on the connecting line between STs within the MSTree indicate the number of different alleles. Solid lines represent an allele difference of three or fewer, whereas dotted lines and faint lines indicate an allele difference of four or more. ESBL, extended-spectrum β-lactamases; MSTree, minimal spanning tree; ST, sequence type.

The sequence type (ST), *bla* genes, and the sampling sites of the ESBL-producing *E. coli* isolates from cats and dogs, respectively, are detailed in Tables 4, 5. MLST analysis identified 20 STs in our ESBL-producing *E. coli* isolates. In total, 16 *E. coli* isolates had STs that did not match any ST in the MLST databank. Combining the data of cats and dogs revealed that the commonest ST was ST457 (13/65, 20.0%), followed by ST131 (10/65, 15.4%), ST648 (6/65, 9.2%), ST38 (3/65, 4.6%), and ST405 (2/65, 3.1%); the other STs were encountered once. ESBL-producing *E. coli* were isolated from several sites but were principally observed in aspirated urine (44/65, 67.7%). Figure 1 reveals the minimal spanning tree of the 65 ESBL-producing *E. coli* STs according to the degree of allele sharing.

**Table 4:** Sequence type, *bla* genes, and sampling site of ESBL-producing *E. coli* in cats.

**ST type**	***bla* genes**
131 (1)^a^	${\text{bla}}_{\text{CTX}-\text{M}-194}^{\text{b}}$
405 (1)	*bla*_CTX−M−194_+${\text{bla}}_{\text{CTX}-\text{M}-124}^{\text{c}}$
457 (5)	${\text{bla}}_{\text{CTX}-\text{M}-55}^{\text{d}}$, ${\text{bla}}_{\text{CTX}-\text{M}-214}^{\text{d}}$, *bla*_CTX−M−55_+*bla*_CTX−M−214_+ ${\text{bla}}_{\text{TEM}-230}^{\text{d}}$, *bla*_CTX−M−55_+*bla*_CTX−M−198_+${\text{bla}}_{\text{TEM}-230}^{\text{d}}$, *bla*_CTX−M−55_+*bla*_CTX−M−198_+${\text{bla}}_{\text{SHV}-199}^{\text{c}}$
648 (3)	${\text{bla}}_{\text{CTX}-\text{M}-124}^{\text{d}}$, ${\text{bla}}_{\text{CTX}-\text{M}-198}^{\text{d}}$, *bla*_CTX−M−55_+*bla*_CTX−M−223_+${\text{bla}}_{\text{TEM}-81}^{\text{e}}$
Unknown (1)	*bla*_CTX−M−55_+*bla*_CTX−M−124_+${\text{bla}}_{\text{CTX}-\text{M}-214}^{\text{c}}$

a *Numbers in parentheses indicate isolation numbers*.

b *From an esophageal feeding tube wound*.

c *From a neck abscess*.

d *From aspirated urine*.

e *From the abdominal cavity*.

**Table 5:** Sequence type, *bla* genes, and sampling site of ESBL-producing *E. coli* in dogs.

**ST type**	***bla* genes**
10 (1)^a^	${\text{bla}}_{\text{CTX}-\text{M}-69}^{\text{b}}$
38 (3)	${\text{bla}}_{\text{TEM}-215}^{\text{c}}$, *bla*_CTX−M−198_+${\text{bla}}_{\text{TEM}-219}^{\text{c}}$, *bla*_CTX−M−198_+${\text{bla}}_{\text{TEM}-215}^{\text{c}}$
69 (1)	*bla*_CTX−M−24_+${\text{bla}}_{\text{TEM}-215}^{\text{d}}$
73 (1)	${\text{bla}}_{\text{TEM}-230}^{\text{e}}$
131 (9)	${\text{bla}}_{\text{CTX}-\text{M}-194}^{2c,f}$, ${\text{bla}}_{\text{CTX}-\text{M}-214}^{\text{g}}$, ${\text{bla}}_{\text{CTX}-\text{M}-174}^{3c}$, *bla*_CTX−M−124_+${\text{bla}}_{\text{CTX}-\text{M}-194}^{\text{c}}$, *bla*_CTX−M−55_+*bla*_CTX−M−67_+${\text{bla}}_{\text{TEM}-215}^{\text{c}}$
359 (1)	*bla*_CTX−M−214_+${\text{bla}}_{\text{TEM}-215}^{\text{c}}$
372 (1)	${\text{bla}}_{\text{CTX}-\text{M}-198}^{\text{c}}$
405 (1)	${\text{bla}}_{\text{CTX}-\text{M}-214}^{\text{h}}$
428 (1)	*bla*_CTX−M−55_+${\text{bla}}_{\text{TEM}-230}^{\text{c}}$
457 (8)	${\text{bla}}_{\text{CTX}-\text{M}-55}^{3c,b}$, ${\text{bla}}_{\text{CTX}-\text{M}-69}^{\text{c}}$, *bla*_CTX−M−55_+${\text{bla}}_{\text{CTX}-\text{M}-214}^{\text{c}}$, *bla*_CTX−M−69_+${\text{bla}}_{\text{SHV}-199}^{\text{d}}$, *bla*_CTX−M−55_+*bla*_CTX−M−214_+${\text{bla}}_{\text{TEM}-230}^{\text{c}}$
636 (1)	${\text{bla}}_{\text{CTX}-\text{M}-55}^{\text{d}}$
648 (3)	${\text{bla}}_{\text{CTX}-\text{M}-198}^{\text{i}}$, *bla*_CTX−M−55_+${\text{bla}}_{\text{CTX}-\text{M}-174}^{\text{c}}$, *bla*_CTX−M−55_+*bla*_CTX−M−148_+*bla*_TEM−215_+${\text{bla}}_{\text{SHV}-199}^{\text{c}}$
1674 (1)	${\text{bla}}_{\text{TEM}-215}^{\text{c}}$
3429 (1)	*bla*_CTX−M−124_+${\text{bla}}_{\text{CTX}-\text{M}-198}^{\text{c}}$
5229 (1)	${\text{bla}}_{\text{TEM}-215}^{\text{c}}$
5640 (1)	*bla*_CTX−M−194_+${\text{bla}}_{\text{TEM}-219}^{\text{j}}$
5685 (1)	*bla*_CTX−M−55_+${\text{bla}}_{\text{CTX}-\text{M}-124}^{\text{k}}$
5686 (1)	${\text{bla}}_{\text{CTX}-\text{M}-55}^{\text{l}}$
5703 (1)	${\text{bla}}_{\text{TEM}-215}^{\text{c}}$
5865 (1)	${\text{bla}}_{\text{CTX}-\text{M}-55}^{\text{c}}$
Unknown (15)	${\text{bla}}_{\text{CTX}-\text{M}-55}^{\text{b}}$, ${\text{bla}}_{\text{CTX}-\text{M}-198}^{\text{c}}$, ${\text{bla}}_{\text{CTX}-\text{M}-211}^{\text{c}}$, ${\text{bla}}_{\text{TEM}-215}^{2c,m}$, *bla*_CTX−M−198_+${\text{bla}}_{\text{TEM}-215}^{\text{c}}$, *bla*_CTX−M−211_+${\text{bla}}_{\text{CTX}-\text{M}-214}^{\text{b},\text{c}}$, *bla*_CTX−M−55_+*bla*_CTX−M−124_+${\text{bla}}_{\text{CTX}-\text{M}-214}^{2\text{c}}$, *bla*_CTX−M−124_+*bla*_CTX−M−214_+${\text{bla}}_{\text{TEM}-226}^{\text{c}}$, *bla*_CTX−M−55_+*bla*_CTX−M−196_+${\text{bla}}_{\text{TEM}-215}^{\text{c}}$, *bla*_CTX−M−124_+*bla*_CTX−M−198_+${\text{bla}}_{\text{TEM}-215}^{\text{c}}$, *bla*_CTX−M−199_+*bla*_CTX−M−124_+*bla*_TEM−215_+${\text{bla}}_{\text{SHV}199}^{\text{n}}$

a *Numbers in parentheses indicate isolation numbers*.

b *From a wound*.

c *From aspirated urine*.

d *From pyometra*.

e *From an oronasal mass*.

f *Pus from paws*.

g *From an abscess*.

h *Pus from left caudal abdomen*.

i *From an ear infection*.

j *From tonsils*.

k *Pus from the esophageal tube*.

l *Pus from intestinal anastomosis*.

m *From a vaginal smear*.

n *From the gallbladder*.

The ESBL-producing *E. coli* isolates from cats were all resistant to ampicillin, ceftiofur, enrofloxacin, and ciprofloxacin, whereas those from dogs were all resistant to ampicillin. All the ESBL-producing *E. coli* were susceptible to imipenem, and more than 50% of the isolates were susceptible to gentamicin. Overall, most strains exhibited a multidrug resistant phenotype (Table 6).

**Table 6:** Antimicrobial susceptibility test of ESBL-producing *E. coli* from dogs and cats.

**Antibiotic discs**	**Cat**, ***n*** **=** **11 (%)**			**Dog**, ***n*** **=** **54 (%)**		
	**Susceptible**	**Intermediate resistant**	**Resistant**	**Susceptible**	**Intermediate resistant**	**Resistant**
Amoxycillin/clavulanic acid	4 (36.4)	1 (9.1)	6 (54.5)	21 (38.9)	14 (25.9)	19 (35.2)
Ampicillin	0 (0)	0 (0)	11 (100)	0 (0)	0 (0)	54 (100)
Imipenem	11 (100)	0 (0)	0 (0)	54 (100)	0 (0)	0 (0)
Ceftiofur	0 (0)	0 (0)	11 (100)	1 (1.9)	3 (5.6)	50 (92.6)
Doxycycline	1 (9.1)	1 (9.1)	9 (81.8)	9 (16.7)	6 (11.1)	39 (72.7)
Enrofloxacin	0 (0)	0 (0)	11 (100)	8 (14.8)	5 (9.3)	41 (75.9)
Ciprofloxacin	0 (0)	0 (0)	11 (100)	11 (20.4)	3 (5.6)	40 (74.1)
Gentamicin	8 (72.7)	0 (0)	3 (27.3)	32 (59.3)	0 (0)	22 (40.7)
Sulfamethoxazole/trimethoprim	3 (27.3)	2 (18.2)	6 (54.5)	25 (46.3)	2 (3.7)	27 (50.0)

**Figure 2:**
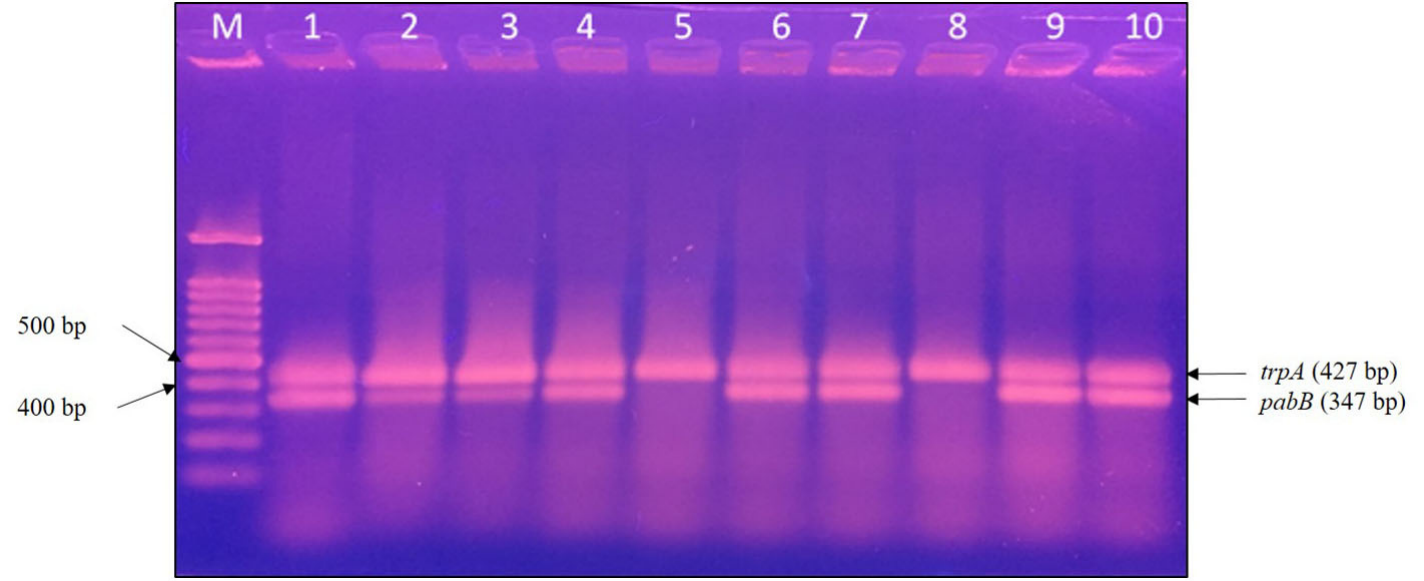
PCR detection of *E. coli* ST131/O25b clone. The *trpA* band corresponds to the positive control in all isolates, whereas the *pabB* band corresponds to the allele-specific amplification obtained only for the ST131/O25b clone. Eight isolates were confirmed to be ST131/O25b clones. M, molecular weight marker, 100 bp DNA ladder; lane 1, *E. coli* 1372; lane 2, *E. coli* 1933; lane 3, *E. coli* 1942; lane 4, *E. coli* 1972; lane 5, *E. coli* 2279; lane 6, *E. coli* 2289; lane 7, *E. coli* 2532; lane 8, *E. coli* 2588; lane 9, *E. coli* 2624; and lane 10, *E. coli* 2670. PCR, polymerase chain reaction.

PCR detection to target *trpA* and *pabB* was performed on 10 *E. coli* ST131 isolates, and 8 isolates were identified as *E. coli* ST131/O25b clones (Figure 2). The ESBL-producing *E. coli* possessed only the *trpA* specific DNA fragment, whereas the ESBL-producing *E. coli* ST131/O25b clones contained both the *trpA* and *pabB* DNA fragments. Among the 10 ESBL-producing *E. coli*, only one ST131/O25b clone was from a cat (*E. coli* 1942), whereas the others were from dogs. The two non-ST131/O25b clones were both from dogs.

## Discussion

The overall prevalence of ESBL-producing *E. coli* in dogs and cats was 23.0% in our study. A comparable prevalence was also reported in Japan, China, and Switzerland (18–20). However, this prevalence is considerably higher than that reported in France (3.7%) and the Netherlands (2%) (21, 22). The medication strategy employed by first-line veterinarians from different countries or regions is a potential explanation for this difference. High prevalence of ESBL-producing *E. coli* threatens the efficacy of third-generation cephalosporins, such as cefovecin, approved for use in veterinary medicine (23).

The *E. coli* isolates were obtained from several sample types in cats and dogs. The most common source of ESBL-producing *E. coli* in cats and dogs was from aspirated urine samples, with prevalence's of 54.5% (6/11) and 68.5% (37/54), respectively. This is unsurprising because urinary tract infection (UTI) is a common diagnosis in companion animals (24). Moreover, UTIs in cats and dogs usually involve a single agent: *E. coli* (25).

The *bla*_CTX−M−1_ _group_ was observed in 58.5% of the *bla* genes. This *bla* gene group is also commonly detected in Europe, the Middle East, and Asia (26). *bla*_CTX−M−55_ was the major *bla* gene in the *bla*_CTX−M−1_ _group_ in our study. CTX-M-15 used to be common in human and animal isolates (27). CTX-M-55 was first identified in Thailand and is closely related to CTX-M-15 with only one amino acid substitution: Ala-77-Val (28). CTX-M-55 is a derivative of CTX-M-15. The presence of CTX-M-55 is widely reported in food and pets in China, and its geographic distribution is primarily in Asian countries (29–31). Notably, CTX-M-55 has rarely been encountered outside Asia. However, the recent emergence of CTX-M-55 in companion animals in Switzerland may indicate the spreading of this enzyme due to international food or animal trade, which warrants further attention (18). A study in the United Kingdom also revealed a decreased prevalence of CTX-M-15 producers over some years in favor of new variants, particularly CTX-M-55 (32). CTX-M-124 was another frequently observed β-lactamase in our study. CTX-M-124 was first detected in wild birds (33); the transmission of CTX-M-124 to other animals from the migratory behavior of wild birds may explain, in part, the presence of CTX-M-124 in ESBL-producing *E. coli* from pets (34).

ST457, ST131, and ST648 are the three major STs of ESBL-producing *E. coli* detected in our study, with ST457 being the most prevalent. This ST has been associated with diseases in companion animals in other studies (21, 35). *E. coli* ST131 and ST648 with CTX-M have been reported worldwide in both human and animal samples. These two clones combine multidrug resistance and virulence; ST131, in particular, is a globally distributed uropathogenic *E. coli* lineage (36). *E. coli* ST131 O25b carrying CTX-M-15 is a globally spreading clone with a high virulence potential, making it a public health concern (37), whereas ST131 O25b with CTX-M-14 has predominated in Japan (38). By contrast, CTX-M-174 and CTX-M-194 were the two main β-lactamases in our *E. coli* ST131 O25b clones. An *E. coli* ST131 carrying CTX-M-174 was identified in humans in Korea (39). CTX-M-174 is a variant of CTX-M-14 with two amino acid substitutions (Glu-7-Leu and Asp-242-Gly). Regardless of the type of CTX-M present in our ST131 isolates, the presence of these clones in cats and dogs raises concerns about potential zoonotic risks. This finding also justifies the continued investigation of ESBL-producing *E*. *coli* to evaluate the persistence of these fast-spreading clones in companion animals in Taiwan. A study in Europe indicated that 1.6% of the diseased dogs and cats carried ESBL-producing *Enterobacteriaceae* but only 2 *E. coli* ST131 isolates were identified; therefore, companion animals may be a source of *bla* genes but may not be the major source of epidemic clones (40).

Previously, LeCuyer et al. (41) revealed a thought-provoking finding regarding uropathogenic *E. coli* in canines. They found that ST372 was the predominant ST in dogs, whereas ST372 was an infrequent human pathogen. The prevalence of ST372 observed in dogs was similar to that of ST131 in human uropathogenic *E. coli* and ST73 in feline *E*. *coli* that caused urinary tract infections. They therefore concluded that each host species may have a particular ST that comprises most of the *E. coli* uropathogens. A French study also reached a similar conclusion, identifying ST372 as the major pathogenic *E. coli* ST in dogs (42). Similar findings in two distinct geographic areas may indicate a dog-specific distribution of pathogenic *E. coli* clones instead of the effect of regional factors (42). In contrast to LeCuyer's and Valat's reports, ST372 was observed only once in our study. Different criteria for the screening of *E. coli* in the study design may have contributed to this discrepancy.

Some STs such as ST3429, ST5229, ST5640, ST5685, ST5686, ST5703, and ST5865, to the best of our knowledge, have not been reported before; therefore, the pathogenic potentials of these strains were unknown.

Imipenem reportedly remains relatively active against ESBL-producing bacteria (43), which is consistent with our results (Table 6). Nonetheless, the use of carbapenems in companion animals should be avoided, since the emergence of carbapenem resistance in companion animals has been reported (44).

The current study had some limitations. AmpC-β-lactamases, which also hydrolyze the third generation of cephalosporins, were not assayed for the *E. coli* isolates. In addition, resistant plasmids were not characterized using PCR-based replicon typing. Although the results obtained in this study originate from only one veterinary hospital, this university-based teaching hospital is the major referral hospital for local veterinary clinics in Taipei. We believe that the information regarding ESBL in cats and dogs reported herein could be helpful for infection management and prevention.

## Data Availability Statement

The raw data supporting the conclusions of this article will be made available by the authors, without undue reservation.

## Ethics Statement

The purpose of collecting these samples from animals was for diagnosis and treatment. An ethical review process was not required for this study according to national/local guidelines.

## Author Contributions

Y-HH conducted the characterization of the phenotype and genotype of the ESBL-producing *E. coli* and drafted the manuscript. N-LK analyzed the ESBL-producing *E*. *coli* through MLST. K-SY conceived and coordinated this research plan. All authors have read and approved the final manuscript.

## Conflict of Interest

The authors declare that the research was conducted in the absence of any commercial or financial relationships that could be construed as a potential conflict of interest.
